# Comparison of the Quality of Orthodontic Treatments Evaluated in Cast and Digital Models According to the ABO-OGS

**DOI:** 10.3390/jcm15010066

**Published:** 2025-12-22

**Authors:** Linda Delgado-Perdomo, Christian Ñustes-Peña, Yegny-Katherine Trillos-Mora, Stephanie Patiño-Méndez, Alejandro Pelaez-Vargas

**Affiliations:** 1 Faculty of Dentistry, Universidad Cooperativa de Colombia, Bogotá 111311, Colombia; linda.delgado@ucc.edu.co (L.D.-P.); cristhian.nustes@campusucc.edu.co (C.Ñ.-P.); yegny.trillosm@campusucc.edu.co (Y.-K.T.-M.); stephanie.patinom@campusucc.edu.co (S.P.-M.); 2 Faculty of Dentistry, Universidad Cooperativa de Colombia, Medellín 055422, Colombia

**Keywords:** ABO-OGS, digital model, analogic model, treatment, orthodontic

## Abstract

The Objective Grading System (OGS) developed by the American Board of Orthodontics (ABO-OGS) provides an objective method to evaluate the quality of orthodontic treatment outcomes. Initially designed to assess individual orthodontists, it is now widely adopted by institutions to evaluate treatment results. However, access to digital cast analysis remains limited in developing countries due to the high cost of specialized software. **Objectives:** This study aimed to compare physical and digital models based on ABO-OGS parameters in finished treatments and to determine the percentage of cases that met the ABO case category specifications in the graduate Orthodontics program at Universidad Cooperativa de Colombia (Bogotá campus) between 2017 and 2021. **Methods:** A retrospective descriptive study analyzed clinical records from 32 patients who completed orthodontic treatment between 2017 and 2021. Standardized plaster casts, digitized casts, and panoramic radiographs were evaluated. Manual assessment was performed using the ABO-OGS gauge on physical casts, while digital assessment was conducted using software on scanned models. Eight ABO-OGS parameters were scored following established guidelines. **Results:** Manual and digital ABO-OGS assessment demonstrated almost perfect agreement. The intraclass correlation coefficient was ICC (A,1) = 0.999 (*p* < 0.0001), and Bland–Altman analysis revealed a negligible mean bias of 0.34 points with narrow 95% limits of agreement (–0.60 to 1.29). Although the Wilcoxon signed-rank test detected a statistically significant difference (*p* = 0.001), the median scores were clinically equivalent (23.0 vs. 23.5). Overall, 69% of cases met the ABO-OGS passing threshold (≤30), while 31% did not (>30). The greatest differences between manual and digital methods were observed in occlusal contacts, marginal ridges, and buccolingual inclination. Occlusal relationships, overjet, and alignment contributed the most to the total ABO-OGS scores. Both linear (Least Absolute Shrinkage and Selection Operator regression—Lasso) and non-linear (Random Forest) models consistently identified the same core predictors, confirming the robustness of digital and manual workflows in capturing key determinants of treatment outcomes. **Conclusions:** Manual and digital methods of ABO-OGS assessment are clinically interchangeable. Despite small statistical differences, digital models provided reproducible results, with 69% of cases meeting ABO-OGS passing criteria. These findings support the validity of digital models as a reliable alternative for orthodontic outcome evaluation.

## 1. Introduction

The orthodontic treatment is focused on improving craniofacial esthetics and function. Around the world, this treatment is provided by accredited orthodontic specialists, who have completed high-level education programs ranging from two to four years. These programs lead to a certificate in orthodontics, or M.Sc. and/or Ph. D. degrees with clinical training. After a century of transition from non-formal to formal orthodontic education, current trends suggest that orthodontics will be one of the most popular dental specialties [1]. When the excellence of the residency program is evaluated, three major domains appear in the surveys: faculty, education, and resident/graduate student/alumni [2]. Other program success indicators include 100% of residents successfully passing the ABO written examination upon graduation and at least 60% of residents achieving full ABO certification within 5 years of graduation. These metrics serve as a robust indicator of the skills provided by a program. However, these criteria are not directly applicable to residency programs outside the United States and Canada. Considering the obvious tendency for harmonization and uniformity, regional differences have been described [3].

Assessing the quality of the final treatment results is a subjective process. Several indices have been developed when searching for objective assessments to quantify the quality of the final result, including the occlusion index, the Peer Assessment Rating (PAR), the Index of Complexity, Outcome, and Need, the Ideal Tooth Relationship Index (ITRI), the Cast-Radiograph Evaluation, and the American Board of Orthodontics-Objective Grading System (ABO-OGS) index [4,5,6,7,8].

In recent years, the ABO-OGS has developed a reproducible index based on the evaluation of eight parameters including alignment, marginal ridges, vestibular–lingual inclination, overjet, occlusal relationship, occlusal contacts, interproximal contacts, and root angulation in the maxillary and mandibular teeth, which is currently described as cast-radiographic evaluation (CRE). Evaluation of the OGS includes the use of panoramic X-rays and analog (plaster) [7] or digital (STL file) or 3D printed dental models [9], following a protocol that enables clinicians to determine whether the outcome of the treatment is approved with scores <20 points or within the approval limit of 20–30 points. Likewise, scores of >30 points are rejected or not approved, thereby creating two classification categories within the index [7].

The ABO-OGS index has been widely adopted as a standardized tool to assess the quality of finishing in institutional orthodontic treatment (e.g., in public hospitals and university clinics [10,11,12,13,14,15]. Notably, several studies have explored the relationship between case complexity and clinical outcomes, comparisons between cases that passed ABO certification and those treated in academic institutions, and the impact of frequent operator changes. In the latter, evidence suggests that this practice is associated with long treatment durations and reduced quality of outcomes [16,17]. However, no significant differences have been reported in the overall score of cases treated in university programs and private practices [18]. It has also been reported that posttreatment outcomes in a graduate orthodontic university clinic showed improved ABO-OGS scores in the years following the implementation of this training [19]. Furthermore, the traditional and simplified ABO-OGS oriented approach has been used to provide a standardized and objective framework for evaluating bracket positioning in undergraduate courses [20].

Reportedly, assessment of physical and digital models has a high correlation in orthodontic diagnosis [21] to the extent that ABO-OGS is currently being implemented for digital models in software such as Ortho Share 3D, SureSmile, OrthoCAD, NemoCast, CS model and Geomagic Qualify. The efficiency of these programs has been validated by different authors [22,23,24,25,26,27]. However, Miranda et al. found a limited correlation between manual and digital methods for alignment, (BL) inclination and occlusal relationship, after evaluation by five different examiners across five sets of models [28] despite the general consensus that automated analysis enhances efficiency and accelerates results generation [24]. Therefore, the aim of this study was to compare physical and digital models based on ABO-OGS parameters in completed treatments, and to determine the percentage of cases meeting ABO case category specifications within the graduate Orthodontics program at Universidad Cooperativa de Colombia (Bogotá campus) between 2017 and 2021. In order to strengthen novelty and analytical robustness in the context of a small sample size—reflecting resource-limited settings, this study employed Least Absolute Shrinkage and Selection Operator (LASSO) regression and Random Forest techniques, leveraging both linear and non-linear modeling approaches.

## 2. Materials and Methods

A retrospective and descriptive study was conducted using a probabilistic sample size and involved the use of dental and panoramic X-ray records. Furthermore, the physical and digitized cast models of the clinical cases treated from 2017 to 2021 were compared. Written informed consent for scientific use of clinical records was obtained from all patients and their legal guardians at the time of registration. This study was approved by the Bioethics Committee for Research at Universidad Cooperativa de Colombia (Code. 012-2019–14 November 2019).

A post hoc sample size calculation was performed for reliability analysis using an online calculator based on the hypothesis testing method for the Intraclass Correlation Coefficient (ICC), as previously reported [29]. The calculation was based on the following parameters: a minimum acceptable ICC (ρ_0_) of 0.80, an expected ICC (ρ_1_) of 0.95, a significance level (α) of 0.05, and a statistical power of 80%, assuming two raters (k = 2; manual and digital) and no expected dropout rate. This analysis indicated that 16 dental casts would have been sufficient; however, 32 casts were included, thereby exceeding this requirement and enhancing the robustness of the results for parametric or non-parametric analyses, as described below.

Post-treatment dental cast models and panoramic radiographs of 32 patients with permanent were included.

Inclusion Criteria:

- Patients with permanent maxillary and mandibular second molars in sound condition.
- No obvious loss of dental tissues due to wear or caries.
- Treatment performed with fixed orthodontic appliances.

Exclusion Criteria:

- Patients who received limited or adjunctive orthodontic treatment.
- Patients with craniofacial syndromes.
- Patients with incomplete records.

Each dental cast model was scanned using the iTero Element Plus scanner (Align Technology, San José, CA, USA) and stored as an STL file with a resolution of 50 µm, following a previously described methodology to obtain the digital model [30]. The ABO-OGS score was similarly determined using the following two methods: the ABO-OGS gauge was used for analog models and the OrthoCAD software (Version 5.9.1.50) was used for digital models. Once the evaluation was completed, the results were exported as a PDF file in accordance with previously reported recommendations [23].

The ABO-OGS index evaluated eight parameters using post-treatment dental casts and panoramic radiographs: alignment/rotations, marginal ridges, buccolingual inclination, occlusal relationship, occlusal contacts, overjet, interproximal contacts, and root angulations. Points were deducted according to ABO standards, with penalties of 1 or 2 points per discrepancy. The third molars were excluded following ABO guidelines. The total ABO-OGS score for each patient was obtained by adding all deductions.

Researchers were standardized to follow the ABO-OGS evaluation protocol using physical and digital records from five patients included in the final analysis. A senior researcher (L. D-P) recorded ABO-OGS parameters twice, with a 2-week interval. Measurements were repeated when discrepancies exceeded two points. Inter-operator analysis was performed by a senior orthodontist (>10 years of experience) and three orthodontic graduate students.

Statistical analyses, including LASSO regression and Random Forest modeling, were performed using custom routines written in MATLAB R2023a (The MathWorks, Natick, MA, USA). Intra- and inter-operator error was quantified using the Dahlberg coefficient. Normality of ABO-OGS score distributions was assessed by the Shapiro–Wilk test. Non-normal data (*p* < 0.05) were compared with the Wilcoxon signed-rank test for paired samples, and the effect size r was computed. Agreement between manual and digital OGS measurements was further evaluated by a two-way mixed-effects, absolute-agreement intraclass correlation coefficient (ICC[A,1]) and by Bland–Altman analysis to estimate mean bias and 95% limits of agreement.

The qualitative classification of results followed ABO-OGS criteria, and cases with an OGS score ≤30 were considered approved, while those with scores >30 were categorized as not approved. Finally, to identify the most predictive parameters of the total ABO-OGS score, both linear and non-linear modeling approaches suitable for small sample sizes were applied. A LASSO regression with ten-fold cross-validation was used as a linear penalized regression method for coefficient selection, while a 100-tree Random Forest with out-of-bag permutation importance was implemented as a non-linear ensemble model to rank predictors.

## 3. Results

Table 1 presents the intra- and inter-operator reliability of the ABO-OGS evaluation based on Dahlberg’s error for both manual and digital methods. The intra-operator error of the senior examiner was minimal and comparable between manual (0.25) and digital (0.20) assessments, demonstrating excellent consistency. In contrast, less experienced examiners exhibited markedly higher intra-operator error values, exceeding 1.60 in both methods. Regarding inter-operator reliability, the manual approach showed greater variability (1.20–1.29) compared with the digital method (0.91–1.04). These findings indicate that while senior examiners maintained consistently low errors regardless of the method, digital evaluation reduced variability among less experienced examiners, supporting its reliability in minimizing operator-dependent discrepancies.

Table 2 shows the comparison of ABO-OGS scores obtained with manual and digital methods. Since both distributions deviated from normality (Shapiro–Wilk test, *p* < 0.05), the Wilcoxon signed-rank test was applied. A statistically significant difference was detected (W = 66, *p* = 0.001, effect size r = −0.654); however, the median scores were nearly identical (23.0 for manual vs. 23.5 for digital), indicating that the difference lacks clinical relevance. The intraclass correlation coefficient confirmed almost perfect agreement between both methods (ICC(A,1) = 0.999, F (31, 31) = 2354.49, *p* < 0.0001), highlighting the robustness of digital scoring in relation to manual evaluation.

Table 2 summarizes the quality of orthodontic treatments according to the ABO-OGS parameters, categorized as ≤30 (approved) or >30 (not approved). The results indicated that 69% of the treatments were classified as approved, whereas 31% did not reach the acceptance threshold. The distribution was identical for both manual and digital methods, underscoring the clinical equivalence of the two approaches. Extreme cases included an approved treatment with only 6 points and a non-approved case with 59 points, illustrating the wide variability in treatment outcomes within the cohort. No differences between methods were observed for alignment and rotations, occlusal relationship, overjet, interproximal contacts, or root angulation. Small discrepancies were identified for marginal ridges (0.03), vestibular–lingual inclination (0.19), and occlusal contacts (0.06). Notably, among cases classified as approved, 69% required a treatment time exceeding two years, highlighting the complexity of achieving high-quality orthodontic outcomes.

**Figure 1:**
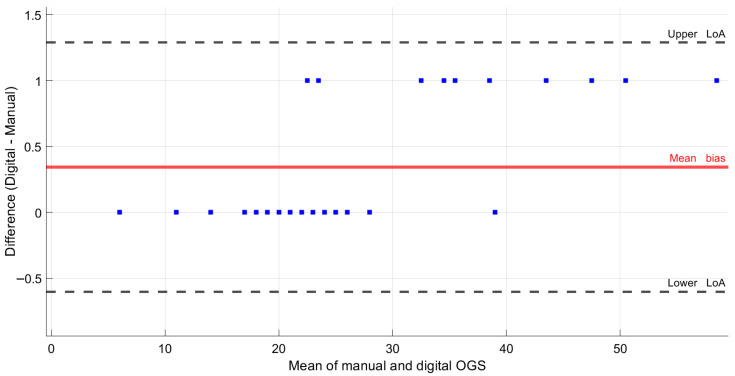
Bland–Altman plot comparing manual and digital OGS measurements. The mean bias between methods was 0.34 points (red line), with 95% limits of agreement ranging from −0.60 to 1.29 (dashed lines). The narrow dispersion of the differences indicates minimal systematic error, supporting the high agreement between both approaches.

Figure 1 exhibits the Bland–Altman analysis, which further supports the high level of agreement between manual and digital assessments. The mean bias was negligible (0.34 points), and the 95% limits of agreement were narrow (–0.60 to 1.29). The restricted dispersion of differences indicates minimal systematic error and reinforces the clinical interchangeability of both approaches. Collectively, these results demonstrate that while digital assessments tend to yield slightly higher values, the difference is clinically trivial. The consistency across intra- and inter-operator reliability, non-parametric testing, ICC, and Bland–Altman analysis highlights the reproducibility of digital models as a robust and valid alternative for ABO-OGS evaluation.

**Figure 2:**
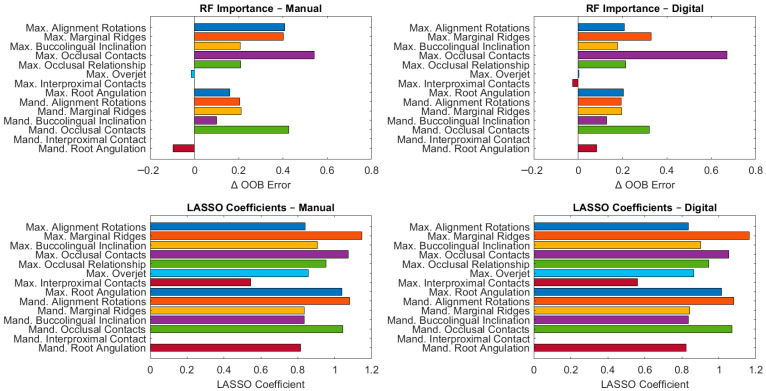
LASSO and Random Forest models for manual and digital ABO-OGS methods. (Max.) maxillary and (Mand.) mandibular.

Figure 2 exhibits the variable selection and predictive modeling results obtained with LASSO and Random Forest analyses. Both approaches consistently identified maxillary marginal ridges, maxillary occlusal contacts, and mandibular alignment/rotations as the strongest predictors of post-treatment ABO-OGS scores, with high LASSO coefficients (≥1.05) and marked increases in out-of-bag error when permuted (RF ΔOOB > 0.25). Conversely, mandibular interproximal contact showed a negligible contribution (LASSO coefficient = 0; RF ΔOOB = 0). Interestingly, maxillary root angulation was retained by LASSO with coefficients > 1.0, but its importance in Random Forest was minimal or negative, suggesting redundancy or multicollinearity. The high concordance between linear and non-linear modeling strategies reinforces the robustness of these predictors and further supports the clinical interchangeability of manual and digital workflows in ABO-OGS evaluation.

Overall, the results consistently demonstrated that digital models provide highly reliable, reproducible, and clinically interchangeable assessments compared with manual scoring. While digital evaluations tend to yield marginally higher scores, these differences, though statistically detectable, are clinically trivial. Furthermore, predictive modeling confirmed that key morphological determinants are robustly captured by both manual and digital workflows, reinforcing the validity of digital models as a strong alternative for ABO-OGS evaluation in orthodontics.

## 4. Discussion

The importance of assessing the quality of finished orthodontic treatments has been previously reported, and many institutions have continuously pursued excellence, as well as harmonization and standardization in graduate orthodontic education. This consideration has been adopted by several dental schools worldwide [10,11,12,13,14,15]. In this study, physical and digital models were compared based on ABO-OGS parameters in finished treatments, and the percentage of finished cases that met the ABO case category specifications was evaluated within our graduate Orthodontics program.

In a retrospective study, Campbell et al. [14] used ABO-OGS to assess the quality of orthodontic finishing from a sample of 382 cases that met the ABO case report category specifications for phase III of the ABO board process. The mean for the entire sample was 32.64 (SD = 13.86). In particular, the study identified a significant impact of early-debonding cases—defined as patients who signed an early-termination consent form to have their appliances removed before ideal treatment outcomes were achieved. When only optimally finished cases were analyzed, the mean score dropped to 29.2 (SD = 11.54). The authors reported that approximately 46% of the cases (≤29 points) might have passed the ABO clinical examination. These reports are clinically relevant due to the substantial sample size and their implications for treatment quality assessment. In the present study, 69% of cases could be approved (≤29 points), although this value was derived from a markedly small sample (*n* = 32), which was used as a baseline to implement strategies for a rigorous case completion protocol and final record verification. This was necessary because many patients did not meet the inclusion criterion of having complete records, as they declined to undergo a new set of diagnostic procedures after debonding. Carvajal-Florez et al. [31] evaluated the implementation of a finishing protocol in university clinics and found that the mean ABO-OGS score was significantly lower in the intervention group (31.4 ± 9.67) compared to the control group (38.0 ± 9.0), indicating improved treatment outcomes [31].

These findings are consistent with those of Vu et al. [32], who reported that after implementing a rigorous finishing protocol, the mean ABO-OGS score was 23.5 ± 11.2 for cases completed over a three-year period (mean = 24.15, SD = 12.48 for 2004; mean = 23.78, SD = 12.71 for 2005; and mean = 22.66, SD = 9.4 for 2006) and 25.19 ± 11.16 for the graduating classes of 2001–2003. The highest values were found for occlusal contacts, buccolingual inclination, occlusal relationships and alignment. These values represent a marked reduction compared with the earlier mean values of 34.36 and SD = 10.39 reported within the same graduate program by Pinskaya et al. [33], based on a sample of 521 patients treated by the graduating classes of 1998, 1999, and 2000. The highest values were found for overjet, occlusal relationships, root angulation and occlusal contacts.

Following this trend, several universities worldwide have studied the percentage of cases potentially achieving a passing ABO-OGS score. The University of Puerto Rico (Puerto Rico) reported a value of 53% in a sample of 64 cases from a study conducted between 2007 and 2008 [11], and Chulalongkorn University (Thailand) reported 98% in a sample of 100 cases from a study conducted between 2017 and 2021 [10]. Cansunar and Uysal [34] found 60% of cases in 1098 patients treated at nine graduate orthodontic programs across various cities in Turkey (Ataturk, Baskent, Cumhuriyet, Cukurova, Dicle, Erciyes, Inonu, Selcuk, and Suleyman Demirel Universities) and they found the highest values for overjet, marginal ridge and occlusal contact variables. The University of Antioquia (Colombia) reported 50% in 40 evaluated cases [13], while Suez Canal University (Egypt) reported 11.5% in 122 evaluated cases [35], the lowest percentage reported until recently in the literature. Song et al. [36] evaluated 108 subjects from a multicenter study in China (Peking University, Sichuan University, Fourth Military Medical University, Capital Medical University, Nanjing Medical University, and Wuhan University) and concluded that ABO-OGS scores greater than 20 points are unacceptable treatment outcomes in a Chinese population. Their mean OGS-ABO score was 19.13 (8.4), with occlusal relationship and overjet being the most predictive components, considering that the root angulation criteria were excluded from the entire study. In 2024, the University of Medical Sciences from Mashha (Iran) reported 36 orthodontic cases that improved from a mean score of 78.5 (SD = 29.79) to a mean score of 18.97 (8.14).

The use of digital technologies in orthodontic practice has increased considerably. Studies have compared manual and digital models to evaluate the quality of orthodontic treatments. In their systematic review, Rossini et al. [9] concluded that digital models are as reliable as plaster models, with high precision, reliability, transfer ease, and reproducibility. Furthermore, with advantages in terms of cost, time, and storage space required, digital models may be considered the new gold standard in current practice. A study by Hildebrand et al. [23] evaluated the ABO-OGS of 36 plaster and digital models using OrthoCAD. The study found high reliability for individual components and the total ABO-OGS score for the digital and analog casts. However, significant mean differences were observed in terms of alignment (0.61 points), occlusal contacts (1.89 points), and overjet (3.94 points). The total ABO score also showed a statistically significant difference (9 points). They concluded that inter-arch measurements were inaccurate in occlusion. In contrast, the present study reported a minimum of 1 point between the manual and digital measurements of various parameters, including marginal ridges, occlusal contacts, and vestibular–lingual inclination. In a study by Okunami et al. [21], 30 plaster models, which were digitized and evaluated using OrthoCAD, were analyzed. This study reported that the mean differences did not exceed 0.05 points for both the plaster and digital measurements. No significant differences were found in the parameters. However, the scores with the highest mean differences were occlusal relationships, and the interproximal scores were the lowest. These results were similar to those observed in the present study owing to the nonsignificant differences between the parameters of marginal ridges, occlusal contacts, and vestibular–lingual inclination.

For patients in the subgroup with outlier ABO-OGS scores (>40), the high scores were attributable to the occlusal relationship, occlusal contacts, overjet, and alignment/rotations, reflecting deficiencies not only in tooth positioning but also in inter-arch coordination. In contrast, interproximal contacts and root angulations had minimal impact, suggesting that the highest scores were driven by a small cluster of functional parameters rather than by diffuse errors across all domains. This pattern indicates that failure to meet ABO approval standards is primarily associated with shortcomings in achieving precise occlusal finishing, underscoring the importance of functional occlusion as a critical determinant of treatment outcomes in this subgroup. This inter-arch parameter may also show greater variability because its refinement occurs mainly during the finishing phase, a stage often constrained by reduced patient compliance and pressure to proceed with bracket debonding before optimal results are achieved. By contrast, intra-arch parameters are typically corrected earlier during the leveling and alignment phase, which may explain their greater stability. Finally, part of the variability might also be related to the proprietary software algorithms, which are not publicly available for evaluation.

To identify the most influential predictors of the total ABO score from the 14 OGS variables, two advanced machine learning techniques were employed: the Least Absolute Shrinkage and Selection Operator (LASSO) regression and Random Forests. Based on a sample size calculation for the Intraclass Correlation Coefficient (ICC) which indicated a requirement of 16 subjects, a total of 32 models were analyzed in this study. This approach was selected over a traditional multiple linear regression analysis, as the latter’s reliance on statistical inference (e.g., *p*-values and confidence intervals) becomes unreliable when the assumption of normally distributed errors is violated—a common occurrence when the response variable itself is non-normal [37]. The selection of these specific methodologies is based on their complementary abilities to address common challenges in regression modeling, such as predictive accuracy and model interpretability. LASSO was selected for its unique ability to perform both coefficient shrinkage and variable selection simultaneously. This process tends to produce a simpler, more interpretable model by setting some coefficients to exactly zero, positioning LASSO as an advantageous intermediate solution that combines the stability of ridge regression with the parsimony of subset selection [38].

On the other hand, Random Forests, an ensemble method that combines multiple tree predictors, was selected for its high predictive accuracy, robustness against overfitting, and more importantly, its ability to perform effectively without making assumptions about the underlying distribution of the data. The accuracy of a random forest depends on the “strength” of the individual trees and the correlation between them; the randomization in selecting features for each node split aims to minimize this correlation while maintaining predictive strength. Using LASSO and Random Forests in conjunction allowed not only for the construction of a robust predictive model but also for a deep and validated understanding of the relative importance of each OGS variable.

This study has several strengths, including the use of a standardized evaluation system (ABO-OGS), the inclusion of both manual and digital methods applied to the same sample, and the incorporation of advanced statistical approaches (ICC, Bland–Altman, LASSO, and Random Forest) that ensured robust reliability and predictive analysis even with a modest sample size. However, certain limitations should be acknowledged. The retrospective design restricts control over potential uncontrolled variables, and the study was limited to a single institution, which may reduce the generalizability of the findings. In addition, the relatively small sample size, although sufficient for the applied statistical methods, may not capture the full spectrum of clinical variability. Future studies should include multicenter cohorts with larger sample sizes to validate these findings, assess longitudinal outcomes, and explore the cost-effectiveness of digital workflows in resource-limited contexts. Furthermore, integrating emerging technologies such as artificial intelligence for automated ABO-OGS scoring may enhance reproducibility and accessibility, particularly in developing countries where specialized software continues to be limited.

## 5. Conclusions

The results showed that digital and manual assessments yielded similar results when evaluating orthodontic treatments using the ABO-OGS.

The quality of the completed treatments evaluated using the ABO-OGS score provided an approval score of 69% of the 32 cases evaluated.

## Figures and Tables

**Table 1:** Intra- and inter-operator analysis according to the Dahlberg coefficient of the ABO-OGS parameters for manual and digital models.

Method	Error	Examiner	Dahlberg
Manual	Intra-operator	Senior	0.25
	Inter-operator	1	1.65
		2	1.20
		3	1.29
Digital	Intra-operator	Senior	0.2
	Inter-operator	1	1.62
		2	0.91
		3	1.04

**Table 2:** ABO-OGS Parameters: Manual vs. Digital Methods.

		Manual					Digital				
	ABO_OGS_Score	Mean	Median	IQR	Min	Max	Mean	Median	IQR	Min	Max
Maxillary	Alignment Rotations	2.6	2	[1.0–4.5]	0	7	2.6	2	[1.0–4.5]	0	7
	Marginal Ridges	1.8	1	[1.0–3.0]	0	5	1.8	1	[1.0–3.0]	0	5
	Buccolingual Inclination	2	2	[0.0–3.0]	0	7	2	2	[0.0–3.5]	0	6
	Occlusal Contacts	2.3	1	[0.0–4.0]	0	9	2.5	1	[0.0–4.5]	0	10
	Occlusal Relationship	5.1	4.5	[2.5–8.0]	0	13	5.1	4.5	[2.5–8.0]	0	13
	Overjet	3.1	2.5	[1.0–5.0]	0	10	3.1	2.5	[1.0–5.0]	0	10
	Interproximal Contacts	0.2	0	[0.0–0.0]	0	2	0.2	0	[0.0–0.0]	0	2
	Root Angulation	1	0	[0.0–1.5]	0	6	1	0	[0.0–1.5]	0	6
Mandibular	Alignment Rotations	2.3	2	[1.0–3.0]	0	7	2.3	2	[1.0–3.0]	0	7
	Marginal Ridges	1.9	2	[0.5–2.5]	0	6	1.9	2	[0.5–2.5]	0	6
	Buccolingual Inclination	1.8	2	[1.0–2.5]	0	5	1.8	2	[1.0–2.5]	0	5
	Occlusal Contacts	1.7	1	[0.0–2.0]	0	8	1.8	1	[0.0–2.0]	0	9
	Interproximal Contact	0.1	0	[0.0–0.0]	0	1	0.1	0	[0.0–0.0]	0	1
	Root Angulation	0.9	0	[0.0–1.5]	0	6	0.9	0	[0.0–1.5]	0	6
	TotalABO	26.7	23	[19.5–33.0]	6	58	27	23.5	[19.5–34.0]	6	59
	comparison (*p* value)	0.001									
Classified		*n*		%			*n*		%		
	≤30 (Approved)	22		69			22		69		
	>30 (Not Approved)	10		31			10		31		
	Total	32		100			32		100		

## Data Availability

The data presented in this study are available on request from the corresponding author.
